# The Utility of Wearable Cameras in Developing Examination Questions and Answers on Physical Examinations: Preliminary Study

**DOI:** 10.2196/53193

**Published:** 2024-07-19

**Authors:** Sho Fukui, Taro Shimizu, Yuji Nishizaki, Kiyoshi Shikino, Yu Yamamoto, Hiroyuki Kobayashi, Yasuharu Tokuda

**Affiliations:** 1 Division of Rheumatology, Inflammation, and Immunity, Brigham and Women’s Hospital and Harvard Medical School, Boston, MA, United States; 2 Department of Emergency and General Medicine, Kyorin University, Tokyo, Japan; 3 Immuno-Rheumatology Center, St. Luke’s International Hospital, Tokyo, Japan; 4 Department of Diagnostic and Generalist Medicine, Dokkyo Medical University Hospital, Tochigi, Japan; 5 Division of Medical Education, Juntendo University School of Medicine, Tokyo, Japan; 6 Department of General Medicine, Chiba University Hospital, Chiba, Japan; 7 Department of Community-oriented Medical Education, Chiba University School of Medicine, Chiba, Japan; 8 Division of General Medicine, Center for Community Medicine, Jichi Medical University, Tochigi, Japan; 9 Department of Internal Medicine, Mito Kyodo General Hospital, University of Tsukuba, Ibaraki, Japan; 10 Muribushi Okinawa for Teaching Hospitals, Okinawa, Japan; 11 Tokyo Foundation for Policy Research, Tokyo, Japan

**Keywords:** medical education, medical technology, wearable device, wearable camera, medical examination, exam, examination, exams, examinations, physical, resident physicians, wearable, wearables, camera, cameras, video, videos, innovation, innovations, innovative, recording, recordings, survey, surveys

## Abstract

To assess the utility of wearable cameras in medical examinations, we created a physician-view video-based examination question and explanation, and the survey results indicated that these cameras can enhance the evaluation and educational capabilities of medical examinations.

## Introduction

Wearable devices have been increasingly used in medicine [1]. Wearable video cameras differ from conventional cameras in that they simulate the perspectives of health care professionals rather than the view of observers. In medical education, wearable video cameras have shown their usefulness in patient interviews [2], virtual physical examination training [3], educational live-streaming ward rounds [4], basic clinical procedures (eg, vascular access) [2], and endoscopic and surgical procedures [5,6]. Wearable cameras, capable of capturing highly realistic situations, can be effective in assessing practical knowledge and providing educational feedback. However, they have not been used in medical examinations. This study aimed to examine the utility of wearable cameras in creating examination questions and answers.

## Methods

### Development of an Examination Question and Its Explanation

**Figure 1.**
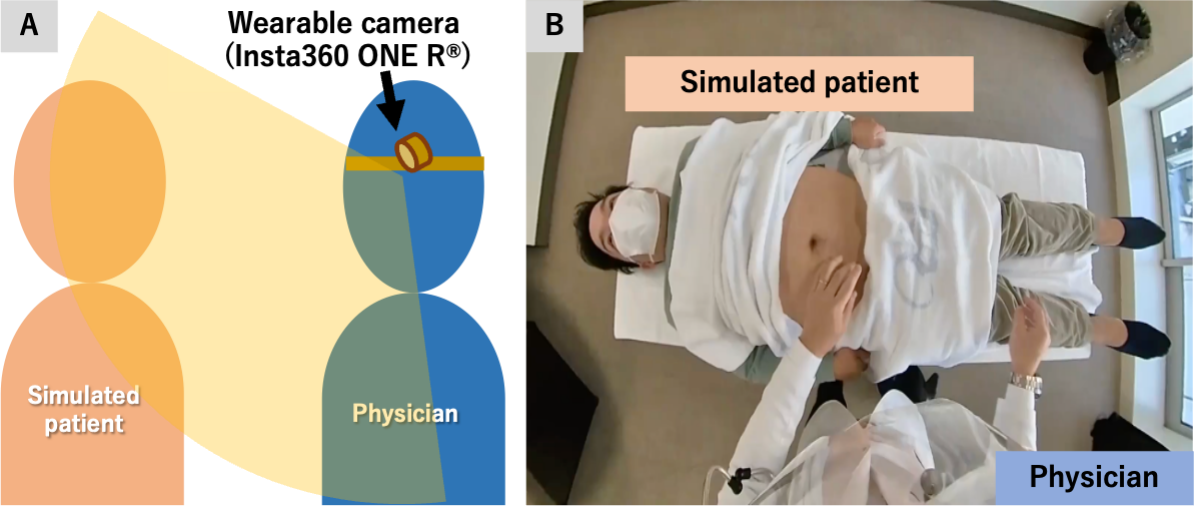
Schematic description of (A) recording physical examination with a wearable camera and (B) its recoded image from the physician’s view.

We developed a single examination question focusing on physical examination skills for resident physicians. In October 2021, authors YN and TS created a simulated outpatient case of appendicitis: a middle-aged man with abdominal pain and localized peritoneal irritation in the right lower quadrant. A volunteer physician played the role of the simulated patient. A physician examined the patient with a wearable camera on his head, recording physician-patient interactions. A compact wide-angle wearable camera (Insta360 ONE R) was used to reproduce a high-resolution physician view, including peripheral view fields (Figure 1).

Using the recorded footage, we created 5 concise videos (about 10 seconds each) depicting various physical examination scenes, including (1) indirect abdominal percussion, (2) checking peritoneal irritation by coughing in the supine position, (3) direct abdominal percussion, (4) the heel drop test, and (5) abdominal palpation; Multimedia Appendices 1, 2, 3, 4, and 5. The examination question asked for the correct sequence of the physical examination. Based on the patient’s position (standing to supine position) and the invasiveness of the examination procedure, 4-2-1-3-5 was considered a correct answer upon the authors’ agreement.

Additionally, we produced an explanatory physician-view answer video in which an experienced physician (TS) explained the proper sequence and key points in abdominal examinations (Multimedia Appendix 6).

### Study Participants, Examination, and Subsequent Survey

The General Medicine In-Training Examination (GM-ITE) for the academic year 2021, a validated nationwide computer-based examination in Japan, was conducted in January and February 2022 [7]. After completing GM-ITE, participants were given the voluntary option to participate in this study on their computer monitors. If agreed, they were requested to answer the question and view the explanatory video. To evaluate the utility of wearable cameras from the examinees’ perspective, participants were asked to complete a subsequent questionnaire survey using the same computers.

### Data Analysis

We described participant characteristics, examination results, and survey results using descriptive statistics.

### Ethical Consideration

We obtained informed consent from the person who played the simulated patient’s role and all other participants before the examination. All data were anonymized, and no honorarium was provided to participants. This study was approved by the Ethics Review Board of the Japan Organization of Advancing Medical Education (approval number: 21‐10).

## Results

A total of 43 resident physicians from multiple Japanese institutions who completed the examination and survey were included. Of these, 28 (65.1%) participants were postgraduate year-1 and 15 (34.9%) were postgraduate year-2 residents; 19 (44%) participants correctly answered the question.

In the postexamination survey, 32 (74%) participants agreed that they could envision real patients better compared to text-based questions (question 1); 26 (61%) were satisfied with the question (question 2); 29 (67%) stated that physician-view videos were more suitable for evaluating clinical competency than observer-view videos (question 3); and 34 (79%) answered that physician-view explanatory video was a more effective educational approach than text-based explanations (question 4; Table 1).

**Table 1.** Results of the survey about the examination and explanatory videos.

Survey questions		Total (N=43), n (%)
**Question 1: Are you able to envision real patients better with this examination compared to a text-based question?**		
	Strongly agree	11 (26)
	Agree	21 (49)
	Neutral	5 (12)
	Disagree	5 (12)
	Strongly disagree	1 (2)
**Question 2: Are you satisfied with this question?**		
	Strongly agree	8 (19)
	Agree	18 (42)
	Neutral	13 (30)
	Disagree	4 (9)
	Strongly disagree	0 (0)
**Question 3: Are physician-view videos more suitable for evaluating clinical competency than observer-view videos?**		
	Strongly agree	13 (30)
	Agree	16 (37)
	Neutral	11 (26)
	Disagree	3 (7)
	Strongly disagree	0 (0)
**Question 4: Is an explanatory video from a physician’s viewpoint more effective for learning the content than traditional text-based explanations?**		
	Strongly agree	13 (30)
	Agree	21 (49)
	Neutral	5 (12)
	Disagree	4 (9)
	Strongly disagree	0 (0)

## Discussion

This study used wearable cameras to create examination questions and subsequent answer explanations; the survey suggested the potential utility of physician-view videos in medical examinations.

This study’s results align with previous research, which showed the effectiveness of chest-mounted point-of-view footage over observer-view videos in physical examination training [3]. For teaching physical examination, video-based e-learning was superior to illustrated text-based e-learning [8]. Moreover, a clinical simulation video successfully assessed clinical competencies across multiple domains in resident physicians [9]. Wearable cameras can provide learners with “immersion,” a sense that one is participating in realistic experiences, which enhances situated learning [10]. Physician-view videos of real clinical situations may emphasize diverse (eg, nonverbal) information. Furthermore, physician-view videos can motivate examinees to learn more actively by regarding themselves as practitioners rather than observers.

This pilot study had limitations. We used a simple subjective survey in a small cohort of volunteer participants. Additionionally, the participants’ detailed characteristics were not collected. More quantitative research with objective outcomes will be required to verify the educational value of incorporating wearable cameras into medical examinations.

## Supplementary material

10.2196/53193 Multimedia Appendix 1 Abdominal examination video 1.

10.2196/53193 Multimedia Appendix 2 Abdominal examination video 2.

10.2196/53193 Multimedia Appendix 3 Abdominal examination video 3.

10.2196/53193 Multimedia Appendix 4 Abdominal examination video 4.

10.2196/53193 Multimedia Appendix 5 Abdominal examination video 5.

10.2196/53193 Multimedia Appendix 6 Answer explanation video.

## Abbreviations

GM-ITE: General Medicine In-Training Examination
